# Thresher Sharks Use Tail-Slaps as a Hunting Strategy

**DOI:** 10.1371/journal.pone.0067380

**Published:** 2013-07-10

**Authors:** Simon P. Oliver, John R. Turner, Klemens Gann, Medel Silvosa, Tim D'Urban Jackson

**Affiliations:** 1 The Thresher Shark Research and Conservation Project, Malapascua Island, Cebu, The Philippines; 2 School of Ocean Sciences, Bangor University, Menai Bridge, Anglesey, Wales, United Kingdom; Aristotle University of Thessaloniki, Greece

## Abstract

The hunting strategies of pelagic thresher sharks (*Alopias pelagicus*) were investigated at Pescador Island in the Philippines. It has long been suspected that thresher sharks hunt with their scythe-like tails but the kinematics associated with the behaviour in the wild are poorly understood. From 61 observations recorded by handheld underwater video camera between June and October 2010, 25 thresher shark shunting events were analysed. Thresher sharks employed tail-slaps to debilitate sardines at all times of day. Hunting events comprised preparation, strike, wind-down recovery and prey item collection phases, which occurred sequentially. Preparation phases were significantly longer than the others, presumably to enable a shark to windup a tail-slap. Tail-slaps were initiated by an adduction of the pectoral fins, a manoeuvre that changed a thresher shark's pitch promoting its posterior region to lift rapidly, and stall its approach. Tail-slaps occurred with such force that they may have caused dissolved gas to diffuse out of the water column forming bubbles. Thresher sharks were able to consume more than one sardine at a time, suggesting that tail-slapping is an effective foraging strategy for hunting schooling prey. Pelagic thresher sharks appear to pursue sardines opportunistically by day and night, which may make them vulnerable to fisheries. Alopiids possess specialist pectoral and caudal fins that are likely to have evolved, at least in part, for tail-slapping. The evidence is now clear; thresher sharks really do hunt with their tails.

## Introduction

Dense aggregations of prey fishes, commonly termed ‘bait balls’, attract large marine predators to areas of high productivity across the globe [1]. Killer whales, *Orcinus orca*, visit fjords in Norway where they use specialist techniques to hunt schooling herring *Clupea harengus* [2], [3], and dolphins are known to migrate through the central Azores for similar purposes [1]. The seasonal run of the sardine *Sardinops sagax* in the nearshore waters of the South African coastline [4] has a strong influence over the abundance and distribution of carcharhinid and lamnid sharks that go there to satisfy part of their diets [5], [6]. In this study it is shown that pelagic thresher sharks, *Alopias pelagicus*, employ specialist techniques to hunt schooling sardines in the waters surrounding a small coral island in the Philippines.

Reaching 365 cm in total length, approximately half of which comprises a scythe-like elongate tail fin, *A. pelagicus* are the smallest of the three recognised thresher shark (*Alopiidae*) species [7]. Described as cosmopolitan sharks that frequent warm and temperate offshore waters circumglobally [8], [9], pelagic thresher sharks mature late, have low fecundity and are classed as ‘vulnerable’ by the International Union for the Conservation of Nature and Natural Resources' (IUCN) Red List [10]. Since the summer of 2010, pelagic thresher sharks have been observed by SCUBA divers to visit Pescador Island in the central Visayas, where they prey upon Indian sardines, *Sardonella longiceps*. Sardines are believed to constitute an important component in the diet of thresher sharks [7] (Oliver unpublished data), and it is proposed that they visit this site to exploit its abundant food resources.

Predation strategies employed by sharks are diverse and vary among species and individuals [11]–[13]. Unique to their taxa, it has long been speculated that thresher sharks use their tails to corral and stun their prey [14]–[16]. Some empirical evidence for this unusual hunting strategy was recently quantified [17] though descriptions of the behaviour remain vague. Under controlled conditions Aalbers et al. (2010) showed that common thresher sharks, *Alopias vulpinus* were able to make contact with tethered bait using their caudal fins. Thresher sharks have also been frequently foul-hooked in the tails by fishermen longlining them [9], [18]. While it has been suggested that bigeye (*Alopias supersiliosus*) and pelagic thresher sharks may employ similar methods of hunting to those described for *A. vulpinus* [7], [17], the kinematics that structure alopiid predatory behaviours in the wild have not been previously documented.

Tail-slapping has been observed in a range of marine predators. Humpback and sperm whales (*Megaptera novaeanglia* and *Physeter catodon*) communicate over great distances with ‘aerial’ tail-slaps [19]–[21], and a similar surface behaviour was described as an agonistic threat to reduce resource competition among white sharks (*Carcharodon carcharias*) in close proximity to each other [22]–[24], though the behaviour was less interpretable when conducted by bait-attracted individuals [25]. Dolphins (*Delphinus delphis*) control the shape and density of schooling prey fish using their tails [1], and killer whales tail-slap bait balls with such ferocity that they produce sound and shockwaves powerful enough to stun fish [2], [3], [26], [27].

When investigating how killer whales forage on schooling herring in Norway, Domenici et al. (1999) showed that tail-slapping enabled the predator to stun up to 33 prey fish with one strike alone. Since sardines school in dense aggregations [4] it can be predicted that thresher sharks employing tail-slaps to hunt them will be able to consume more than one prey item at a time.

In this paper, evidence is provided to show that pelagic thresher sharks use their tails to prey upon sardines, and the kinematics associated with the behaviour are investigated. Hunting events were quantified from handheld video observations to address the following hypotheses: (1) thresher sharks execute a series of rapid body motions that drive tail-slaps during hunting events; (2) tail-slapping enables thresher sharks to stun several prey items at a time. Thresher shark hunting behaviour is discussed in relation to kinematics and hydrodynamics.

## Methods

All of the research (including the handling of marine life, and the interruption of shark behaviour) was undertaken with the permission of the Governor of Cebu and adhered to the Philippine ‘Wildlife Resources Conservation and Protection Act’. The handling of marine life complied with Bangor University's *Research Ethics*' framework and ethical policy, and was approved by the College of Natural Sciences' Animal Ethics Committee.

### Location

**Figure 1:**
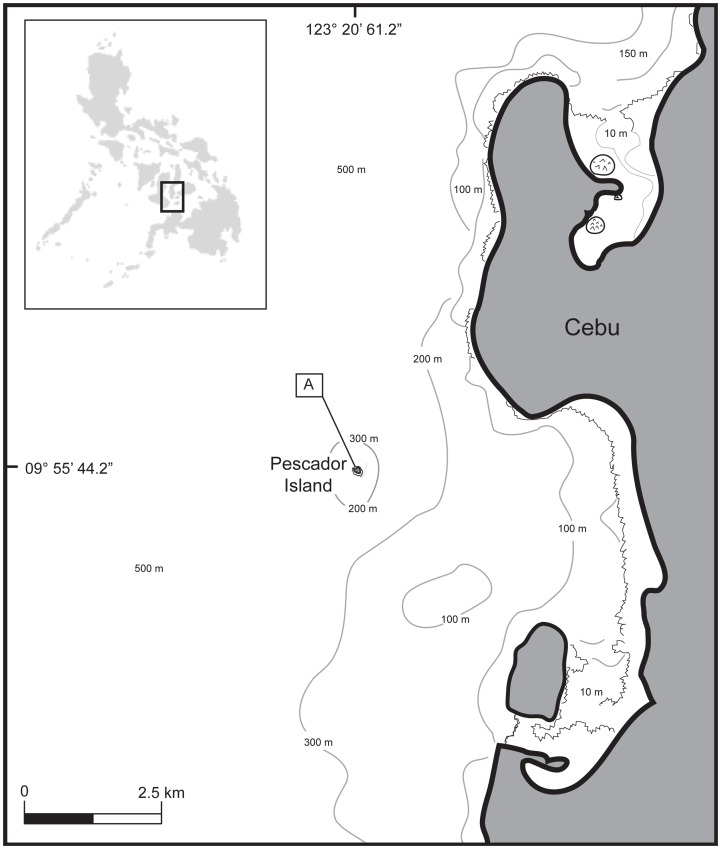
Map showing the location of Pescador Island off Moalboal, Cebu, in the Philippines. A dense aggregation of sardines *Sardonella longiceps*, can be observed year round along the northwestern crest of the fringing reef (A) where thresher sharks hunt them.

Pescador is a small coral island situated in the Tañon Strait (N 09° 55′ 44.2′, E 123° 20′ 61.2′), approximately five kilometers due west from Moalboal, Cebu, in the Philippines (Figure 1). The island is fringed on all sides by a coral reef formed by a shallow plateau of low profile *Acropora* that crests and sheers down 60 m to the sea valley below. Although fishermen have exploited its resources for decades, Pescador's marine biomass is rich and recreational divers visit the island to observe its diverse wildlife on most days, generating important income for the region. A dense aggregation of sardines *Sardonella longiceps,* which can be observed year round along Pescador's northwestern reef crest, attracts a variety of marine predators including pelagic thresher sharks (Figure 1).

### Sampling

Fieldwork was undertaken over 70 days, spanning five months, from June to October 2010 (fieldwork was time restricted due to resource limitations). Handheld underwater video cameras were used by SCUBA divers to record thresher shark hunting behaviour during hour-long dives conducted between 09:00 and 16:00 hours. SCUBA divers used Sony Camcorders® FX-1 and HVR-Z1 housed in Gates Z1 underwater housings, fitted with dome ports, with their focal ranges locked to 0.4 m, and recorded their observations of thresher sharks onto MiniDVs in 1080i 50 (25 fps^−1^) and 1080i 60 (29.97 fps^−1^) HDV formats. Video records were captured opportunistically in the water column between 10 and 25 m depths with the camera recording when a thresher shark was present and observable in the viewfinder. Recordings were downloaded to a hard drive and screened for analysis.

On some occasions, divers interrupted the feeding behaviour of the sharks to collect stunned and dead sardines by hand from the water column. These were brought to the surface where they were inspected for injury, photographed, total length measured, and then released if they were alive. Observable injuries sustained by collected individuals were assumed to be associated with a thresher shark's predatory behaviour. Since no stunned or dead sardines were observed prior to thresher shark attacks, their presence in the water column was used as a proxy for a successful hunting event.

### Analysis of Video Recordings

**Figure 2:**
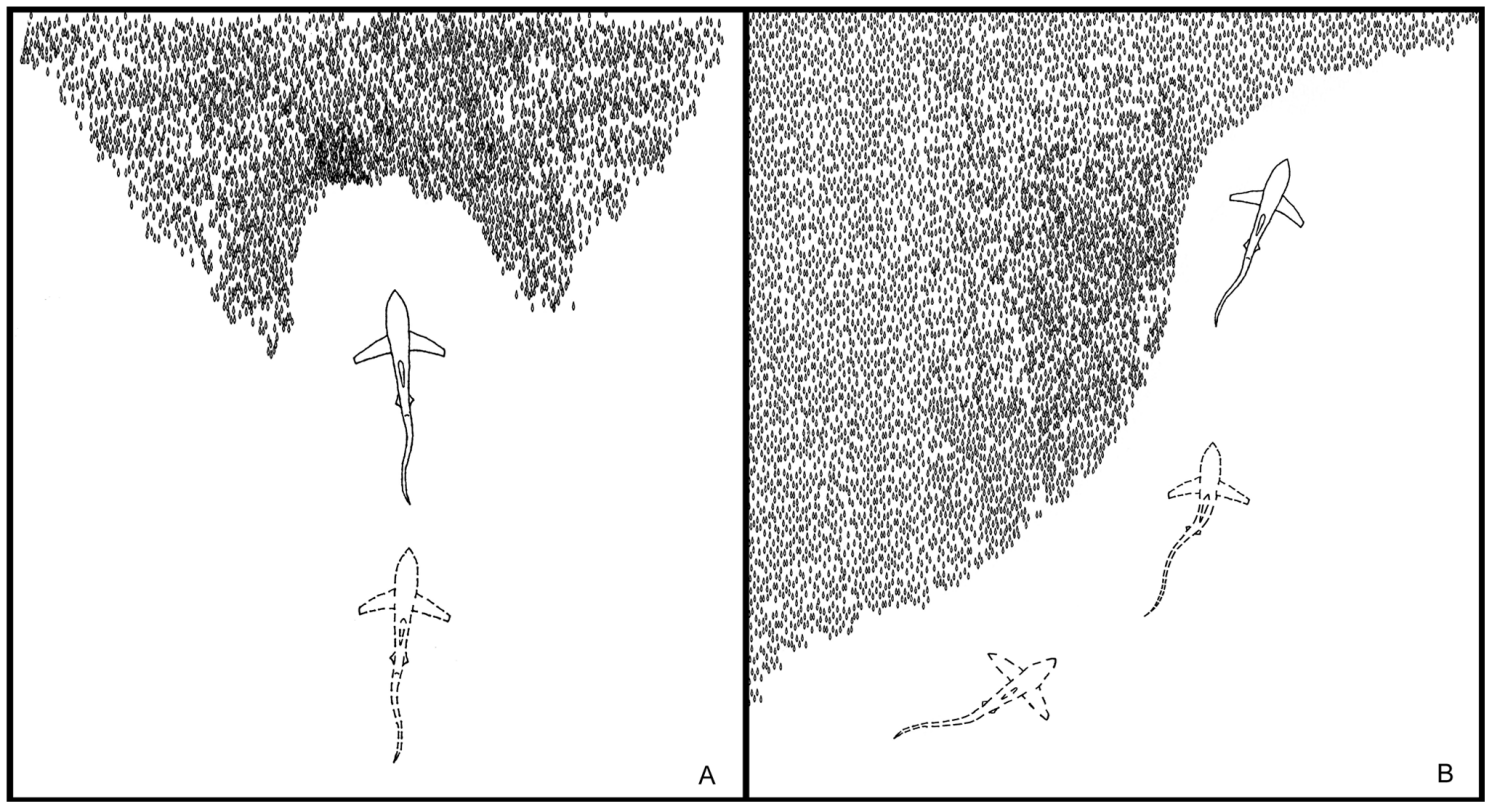
Diagram showing a thresher shark's position relative to the bait ball during (A) overhead; and (B) sideways tail-slaps.

Video sequences documenting thresher sharks' hunting behaviour were classified into two main event types: those in which predation attempts were characterised by (1) an overhead tail-slap or (2) a sideways tail-slap. Overhead tail-slaps typically took place when the shark was positioned perpendicular to and facing the perimeter of the bait ball (Figure 2-A). Thresher sharks slapping at sardines from the side while they were aligned parallel to them characterised ‘sideways tail-slaps’ (Figure 2-B).

### Analysis of Thresher Shark Behaviour

For analysis, hunting events were partitioned into ‘phases’ that were characterised by observable changes in a thresher shark's movement and behaviour during a tail-slap. Termed ‘preparation’, ‘strike’, ‘wind-down recovery’ and ‘prey item collection’, phases were analysed in 25 or 29.97 frames s^−1^ resolution using *Final Cut Pro 7* (Apple Inc., CA) to document behaviours, and video still images were used to construct diagrams [28]. Examples of the video data are available in the supporting information (Movies S1–S4).

### Defining Terms and Classifying Behaviours

The terms ‘motion’, ‘mechanics’ and ‘kinematics’ were adapted from their standard uses in the literature [12]. Motion referred to a change in position of the anatomical structures involved in a thresher shark's hunting behaviour (mouth, caudal peduncle, tail) with respect to time and a fixed reference point. Mechanics referred to the functioning of the anatomical structures and considered the motions of the various parts, as well as the forces acting against them. Kinematics referred to the analysis of the motion alone without reference to any counter forces.

Protocols developed by Slater for categorising behaviour [29] were used to differentiate the behavioural patterns observed in thresher sharks as they preyed upon sardines. A shark's relative orientation (parallel or perpendicular to the bait ball) and the kinematics of the mouth, caudal peduncle and tail were used to compare behaviours between the phases of the event types.

### Shark Length

To estimate a thresher shark's length measurements (total length (TL); precaudal length (PCL); and dorsal caudal fin margin (CDM)), a still image was taken from its video record when the shark was planar to, or in contact with, one of the sardines it was hunting, and both were perpendicular to the axis of observation. Assumed to be equal to the mean (± SE) of the total lengths (cm) of the sardines collected by SCUBA divers *in situ* (11.588±0.142, n = 56), the total length of a referenced sardine was measured in pixels using an image histogram in *Photoshop CS4* (Adobe, San Jose, CA). Lengths were then measured in pixels for the thresher shark in the still image. The shark's actual lengths (cm) could then be expressed as

where (f) was shark, (p) pixels and (s) the referenced sardine. Shark sex was determined by the presence or absence of claspers.

### Kinematics

Only sagittal and transverse plane video observations were selected for kinematic analysis, in which all four phases of the tail-slaps occurred within full view of the camera, and where the shark was close enough to identify the key anatomical parts used for hunting. Of the 22 recordings of overhead tail-slaps, only six sagittal and two transverse plane events were considered suitable for analysis. None of the video records of the sideways tail-slaps met selection standards and were therefore only used to describe the behaviour.

**Figure 3:**
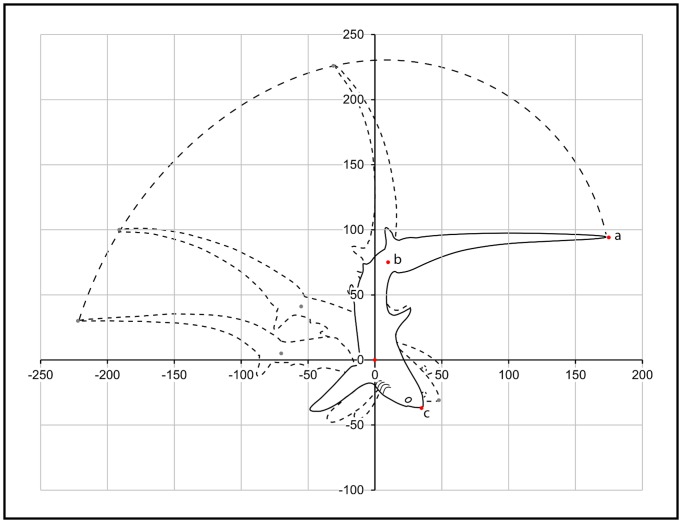
Diagram showing the method used for analysing the kinematics of a thresher shark's tail-slap from a sequence of video still images. For sagittal plane events, three key anatomical parts (a) the tip of the tail, (b) the midpoint of the caudal peduncle, and (c) the tip of the snout were tracked in two dimensions using the posterior base of the pectoral fin as a fixed reference point (x/y intercept  = *0*). The arc length of a thresher shark's tail-slap is shown in dashed line.

For sagittal plane events, three key anatomical parts (i) the terminal caudal fin lobe (tip of the tail), (ii) the midpoint of the caudal peduncle, and (iii) the tip of the snout were tracked in two dimensions by analysing a sequence of video still images. Using the posterior base of the pectoral fin as a fixed reference point, the coordinates of the anatomical parts were plotted for each still frame (Figure 3). Coordinates were expressed as the actual distance (cm) each part was from the base of the pectoral fin (x/y intercept  = *0*) at the time it was plotted, and referenced in degrees, as well as by the speed it was travelling (ms^−1^). Anatomical parts were only plotted when they were clearly visible in the video still images. All video still images were oriented with left to right movement for analysis.

During the peak accelerations of the strike phase, the speed with which the tip of the tail travelled exceeded the frame rate of the underwater cameras used to record it. As a result, some images of the terminal caudal fin lobe were blurred, for all of the selected recordings. To plot the coordinates of the blurred images, a still image taken from a point in the video sequence when the terminal caudal fin lobe was clearly observable was layered on top of the original still image. The leading edge of the caudal fin, the caudal notch and the lower caudal lobe for the two layered still images were aligned. Since all of the leading edges of the caudal fins aligned precisely and only the tips of the caudal fins were blurred for all occurrences, it was assumed that the position of the terminal caudal fin lobe would not alter, relative to its orientation in the strike phase, and its coordinates were plotted from the image layered on top.

For transverse plane events, the pectoral fins were the only anatomical features to be tracked. The ventral midpoint between the pectoral fins was used as a fixed reference point, and coordinates for both the tips of the pectoral fins and their posterior bases were plotted for each video still frame. The angles at which the pectoral fins protruded from a shark's body were measured and the angular velocity with which they adducted to initiate a tail-slap was calculated using trigonometry.

### Statistical Analysis

All statistical analyses were carried out in *GenStat 8.1* (Rothamsted Experimental Station, Harpenden, UK) and *Minitab 16* (Minitab Inc., State College, PA, USA). To compensate for the relatively low resolution used to record thresher shark hunting events (25 or 29.7 fps^−1^), a spline curve was used in SigmaPlot Version 11 (Sytat Software, Inc., Hounslow, London, UK) to smooth the plotlines of the graphs given in the figures.

Tail-slap arc lengths (AL) (Figure 3) could not be measured for all of the sagittal plane events because in some of the video observations tail-slaps took place at an angle that was not perpendicular to the camera, while in others they were partially obscured by the sardines. Yet for all events (n = 16) a thresher shark's precaudal length and the duration of its strike phase could be documented. When considering only the events in which a tail-slap's arc length could be measured (n = 6), least squares linear regression analysis was used to compare the distance the tip of a thresher shark's tail travelled during its strike phase, with the shark's precaudal length. PCL could then be used to predict the arc lengths for events in which they could not be directly measured (n = 10), by employing the resulting regression equation

To generate conservative estimates of the speed with which the tip of the tail travelled, straight-line distances between the plotted coordinates were used for calculations, even if the path of the coordinates formed an arc-like motion. Time was expressed as the number of video frames it took for a motion to be completed divided by the frame rate used by the camera upon which the observation was recorded (25 or 29.97 frames s^−1^). Speed was calculated by dividing the straight-line distance between the coordinates of the motion by time (ms^−1^). Speed was only calculated for the motion of the tail, and not the forward locomotion of the shark.

Least squares linear regression analysis was used to examine if tail-slap speeds (max and mean) were related to a shark's size (PCL), and/or the length of its tail (CDM). A tail-slap's maximum speed could only be calculated from the six sagittal plane events for the regression, since frame-by-frame analysis of the arc, which formed the tip of the tail's travel path, was required. The times it took for the tip of the tail to reach maximum speed and arc height were standardised as a proportion of the duration of the strike phase, and compared using a paired *t* test.

To assess the trajectory of a thresher shark's tail-slap, strike phases were standardised for all sharks by dividing the arc that formed the travel path of a thresher shark's tail-slap into nine equal time segments, with one trajectory angle measured for each (Figure 3). A two-way analysis of variance was then used (with trajectory angle as the response variable and shark and time segment as treatments) to test for differences between them.

To examine if a phase's duration varied by the size of a shark, a two-way analysis of variance (with duration as the response variable and PCL and phase type as treatments) was used. The variability between the duration of the different phase types (preparation, strike, or wind-down recovery) was also investigated (one-way ANOVA with duration as the response variable and phase type as treatment).

The relative amplitudes of the movements (defined as the vertical distance between the lowest and highest points attained by a tracked anatomical part) of the tip of a thresher shark's tail, its snout and its caudal peduncle were compared among preparation, strike and wind-down recovery phases for the sagittal plane events, by using a two-way ANOVA (with amplitude as the response variable and phase type, anatomical part and associated interactions as treatments).

## Results

### Recorded Events

A total of 25 thresher shark hunting events were recorded at all times of day (09:00 to 16:00 hours; June – October 2010), 22 of which were overhead tail-slaps. Although divers observed sideways tail-slaps *in situ* on six separate occasions, only three were recorded on video. Both types of event took place over several seconds and involved (i) preparation; (ii) strike; (iii) wind-down recovery; and (iv) prey item collection phases, which occurred sequentially. Six video observations of overhead tail-slap events were not considered for analysis either because the sardines obscured them, the duration of the strike phase could not be documented, or because they occurred at too great a distance from the camera. Precaudal lengths for thresher sharks in the remaining 16 overhead events had a mean (± SD) of 154.67±31.58 cm (Table 1).

**Table 1:** Length measurements (cm) of 16 pelagic thresher sharks, as determined from video records of overhead tail-slap hunting events (June – October 2010).

Sex	PCL	CDM	FL	TL
Unknown	97.20±8.93	90.85±8.35	106.16±9.75	188.05±17.27
*Male*	*104.17*±*9.57*	*110.35*±*10.14*	*113.86*±*10.46*	*214.52*±*19.71*
*Male*	*130.27*±*11.97*	*135.16*±*12.42*	*148.53*±*13.64*	*265.43*±*24.38*
*Female*	*130.29*±*11.97*	*133.24*±*12.24*	*148.08*±*13.60*	*263.53*±*24.21*
*Female*	*134.92*±*12.39*	*124.37*±*11.42*	*148.56*±*13.65*	*259.29*±*23.82*
Female	136.14±12.51	118.61±10.90	147.96±13.59	254.75±23.40
Female	142.12±13.05	136.68±12.56	158.25±14.54	278.80±25.61
Female	153.77±14.13	167.06±15.35	167.93±15.43	320.82±29.47
Unknown	157.37±14.46	158.23±14.53	170.87±15.70	315.60±28.99
*Male*	*161.50*±*14.84*	*160.25*±*14.72*	*176.15*±*16.18*	*321.75*±*29.56*
Male	173.51±15.94	179.38±16.48	189.78±17.43	352.89±32.42
Male	184.95±16.99	161.77±14.86	202.82±18.63	346.72±31.85
Female	186.82±17.16	219.03±20.12	197.37±18.13	405.86±37.28
*Male*	*188.61*±*17.33*	*184.12*±*16.91*	*210.10*±*19.30*	*372.73*±*34.24*
Male	195.61±17.97	218.99±20.12	215.90±19.83	414.60±38.08
Male	197.52±18.14	138.59±12.73	218.20±20.04	336.11±30.87

Lengths were calculated from video images by counting the number of times a referenced sardine could fit lengthwise into the selected length measurements of a thresher shark. Precaudal (PCL), dorsal caudal fin margin (CDM), fork (FL) and total (TL) lengths are presented±their standard deviations. The six sagittal plane events, which were selected for kinematic analysis, are in italics.

**Figure 4:**
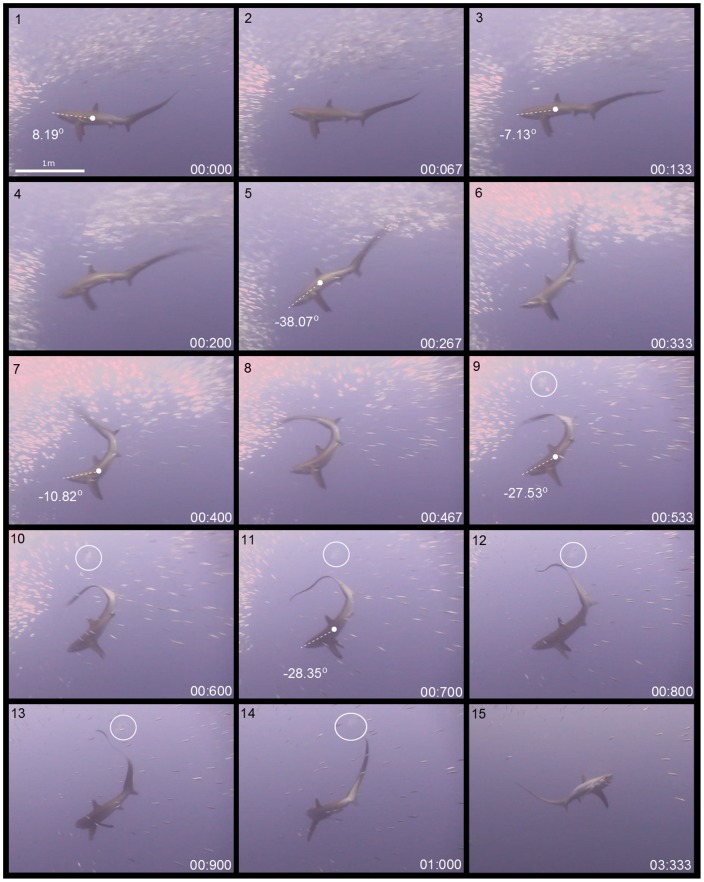
A sequence of still images taken from an overhead tail-slap hunting event that occurred in the sagittal plane (Movie S1). A thresher shark lunged at the bait ball in the horizontal plane (1–3). It then adducted its pectoral fins in a manoeuvre that changed its pitch, promoting its posterior region to lift rapidly and stall its approach (4–6). After adducting its pectoral fins, the shark rotated them laterally in a surge to counter the momentum of its body from precipitating forward (7–10). A rapid and powerful ventro-dorsal peduncular motion drove its tail from its base in a trebuchet catapult motion that terminated overhead in a slap (7–10). The tail-slap occurred with such force that it caused dissolved gas to diffuse out of the water column forming small bubbles that entrained and grew in size (circled in 9–14). The shark returned its pitch to the horizontal plane in a wind-down recovery (11–14), turned 180°, and proceeded to collect the five sardines it had stunned (15). The center of mass about which the movements associated with the shark's overhead tail-slap occurred, changes in camber and time stamps are shown in white.

**Figure 5:**
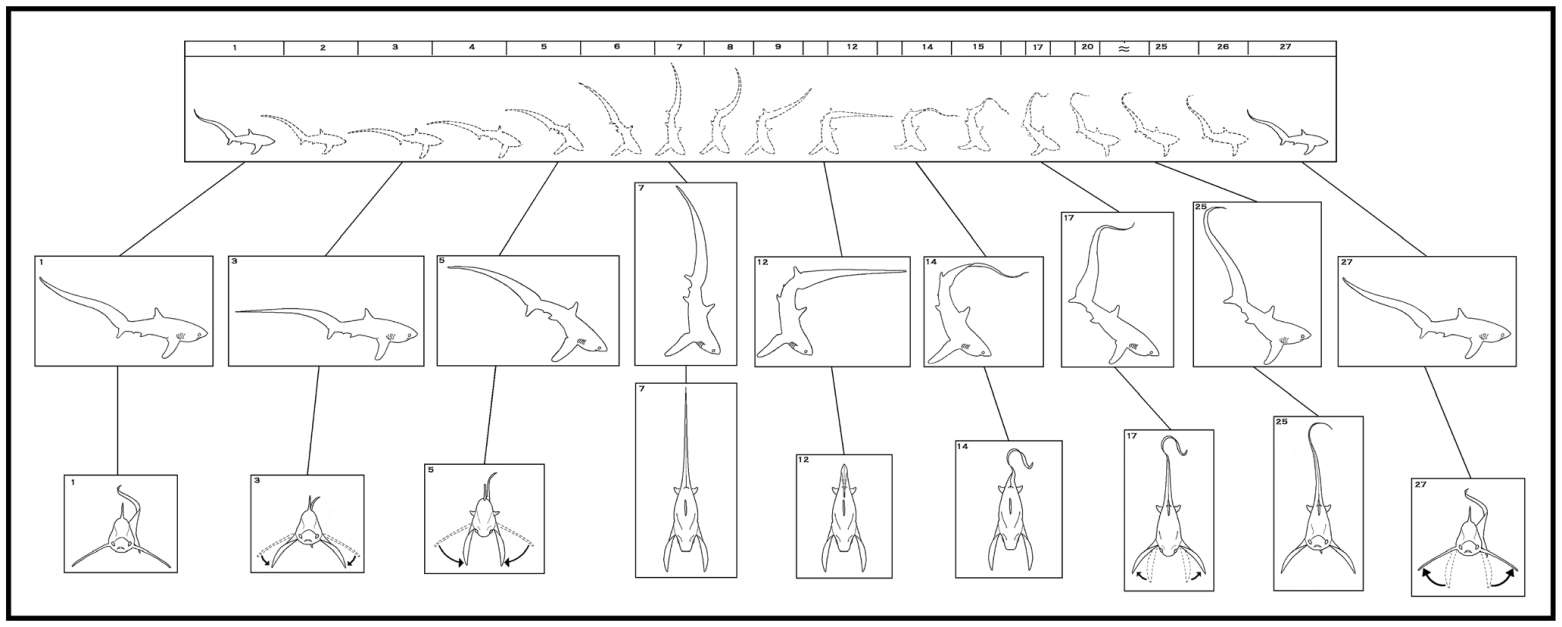
Behaviour diagram of a thresher shark's overhead tail-slap, with preparation (1–2), strike (3–14) and wind-down recovery (15–27) phases, as observed from events, which occurred in the sagittal plane. A motion animation (top) represents 1.08 s^−1^ of an event which was recorded by handheld underwater video camera on 17 June, 2010. Center inserts profile the key characteristics of the behaviour, while inserts shown in the transvers plane (bottom), were interpreted from other video sequences.

**Figure 6:**
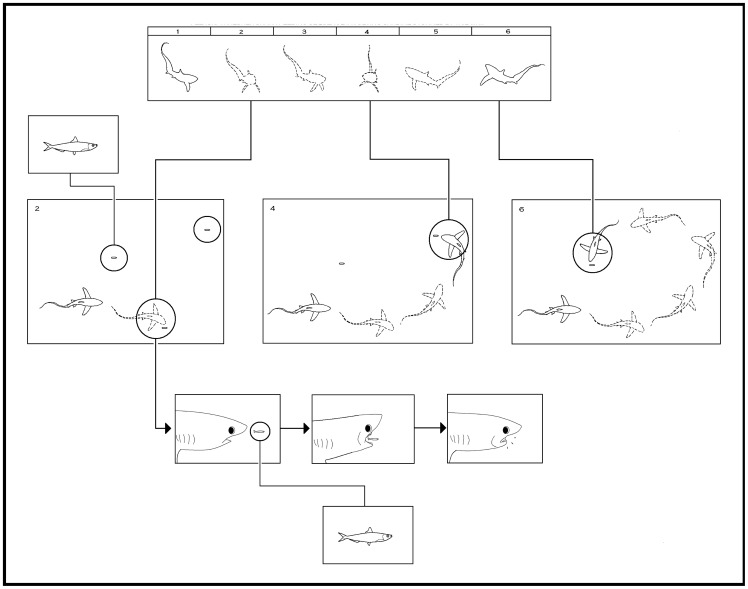
Behaviour diagram of prey item collection phase, as observed from events that were recorded in the sagittal plane. A motion animation (top) represents 3.16 s^−1^ of an event that was recorded by handheld underwater video camera 19 August 2010. Inserts show a thresher shark circling and collecting three sardines that were stunned during the strike phase of a successful hunting event.

Event phases were defined by observable changes in the speed, vertical motion and directional orientation of the tracked anatomical parts, in particular, the positioning of the tip of the tail. A thresher shark accelerating in a lunge towards the bait ball characterised preparation phases. Strike phases were characterised by a slap of the tail. Strikes began with a shark adducting its pectoral fins, a manoeuvre that changed the shark's pitch promoting its posterior region to lift rapidly, and stall its lunge approach. The shark's tail then accelerated in a whip as it travelled overhead the length of its body to the tip of its snout (Figures 4, 5). The wind-down recovery phase involved a thresher shark returning the anatomical parts of the hunting apparatus to their original state. The final prey item collection phase was typically characterised by a thresher shark turning 180**°** and collecting dead and/or stunned sardines if the predation attempt was successful (Movies S1, S2) (Figure 6) or not collecting sardines if it was not (Movie S3).

**Figure 7:**
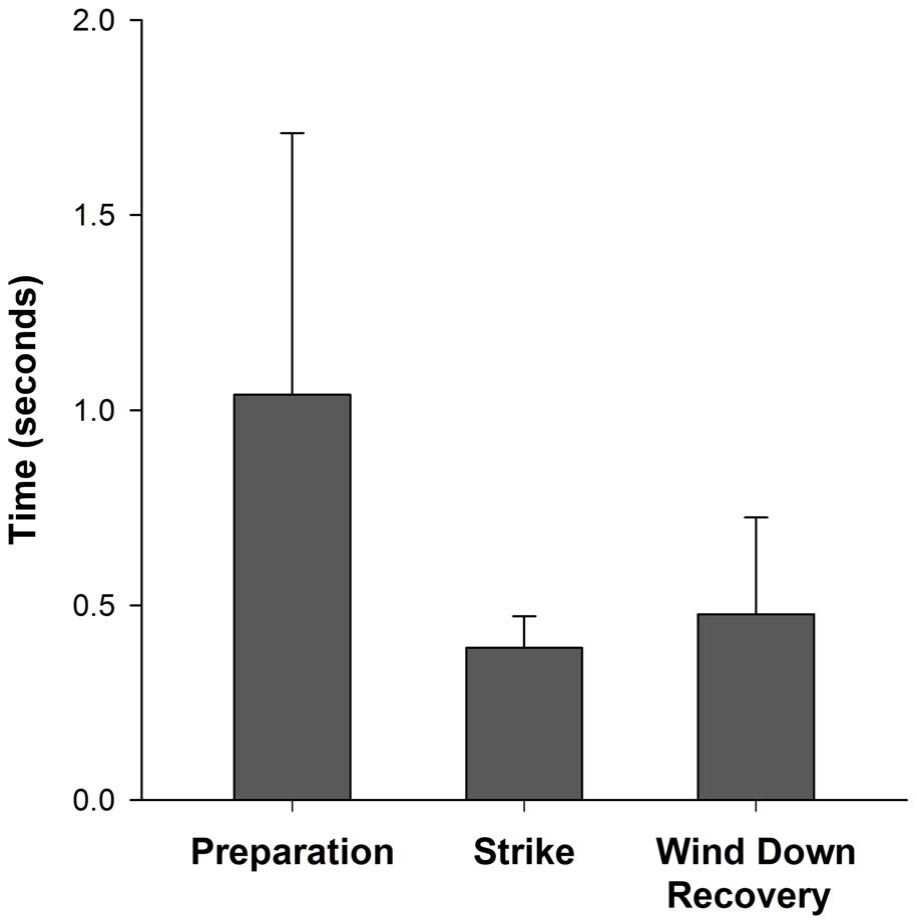
Preparation, strike and wind-down recovery phase durations±their respective standard deviations (SD).

When considering only preparation, strike and wind-down recovery phases, the mean (± SE) duration for overhead tail-slaps was 1.91±0.19 seconds (95% CI: 1.54–2.28 seconds). The preparation phase lasted significantly longer than the strike and wind-down recovery phases (f_2,45_  = 11.53, p < 0.0001) (Figure 7), but there was no correlation between phase duration and shark size (f_2,30_  = 1.14, p = 0.368). The longest recorded event lasted 3.40 seconds and comprised 102 still video images; the shortest lasted 1.13 seconds and comprised 34 video still images.

Of the 16 events considered suitable for analysis, 15 involved a thresher shark turning 180**°** at the end of the wind-down recovery phase**,** presumably to collect dead and/or stunned sardines (Movies S1–S4). Feeding on sardines was observed during the prey item collection phase of five events. These were categorised as successful (Figures 4, 6; Movies S1, S2). The mean (± SE) number of fish consumed by a thresher shark during successful events was 3.60±0.87 sardines. The most successful event resulted in a thresher shark consuming seven sardines, and the least of the successful events resulted in the consumption of two.

### Tail-Slap Kinematics

**Figure 8:**
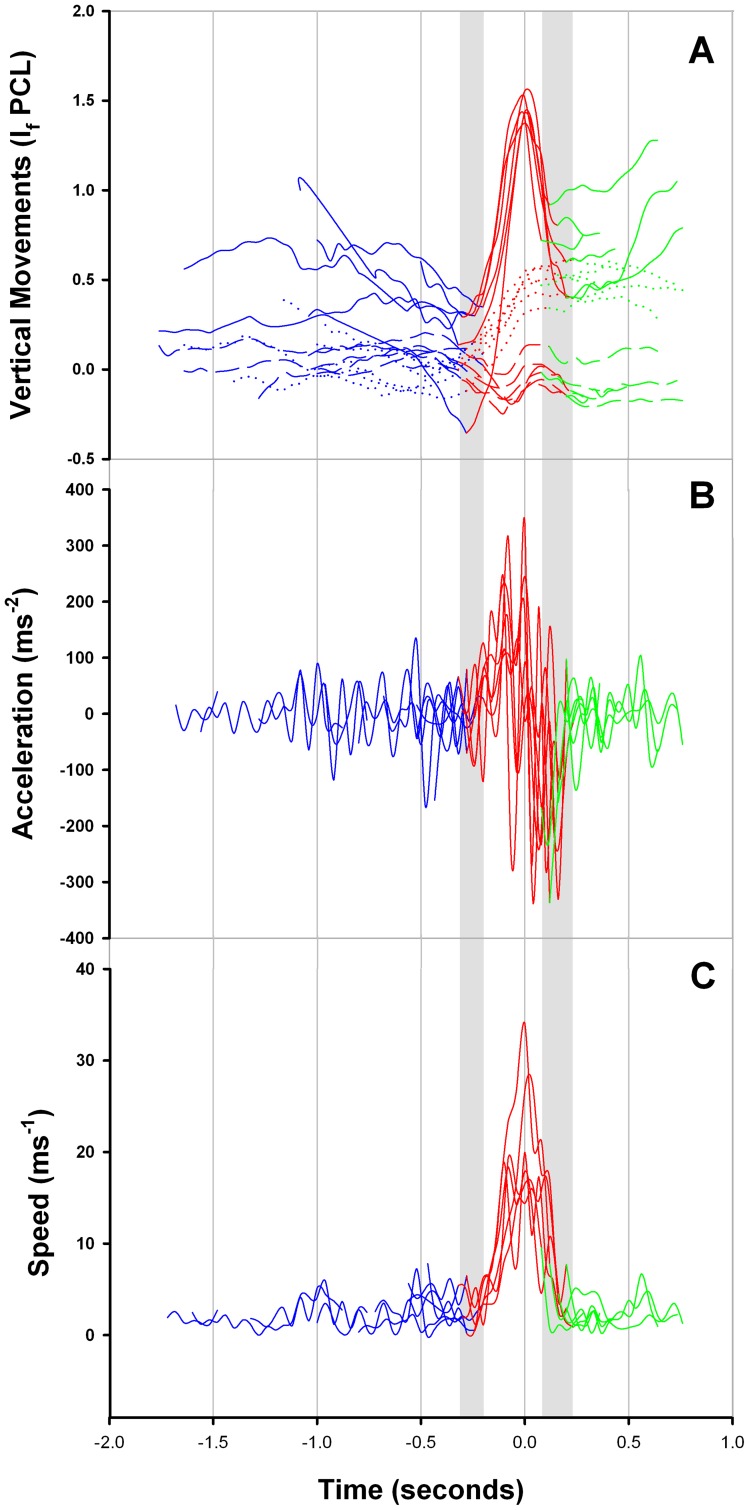
The kinematics of an overhead tail-slap, as observed from six thresher shark hunting events that were recorded in the sagittal plane. A) The movements of the tip of the tail (solid), the caudal peduncle (dotted) and the snout (dashed) were tracked in relation to their relative distances (cm) from the posterior base of the pectoral fin (x/y intercept  = *0*) at the time they were plotted. B) The biphasic acceleration of the tip of a thresher shark's tail reached its peak at the apex of its trajectory arc. C) The maximum trajectory speeds of a thresher shark's tail were tracked in relation to their relative distances (cm) from the posterior base of the pectoral fin (x/y intercept  = *0*) at the time they were plotted. The variability in the separation points of preparation (blue); strike (red); and wind-down recovery (green) phases among the six analysed events is shown in grey shading.

**Figure 9:**
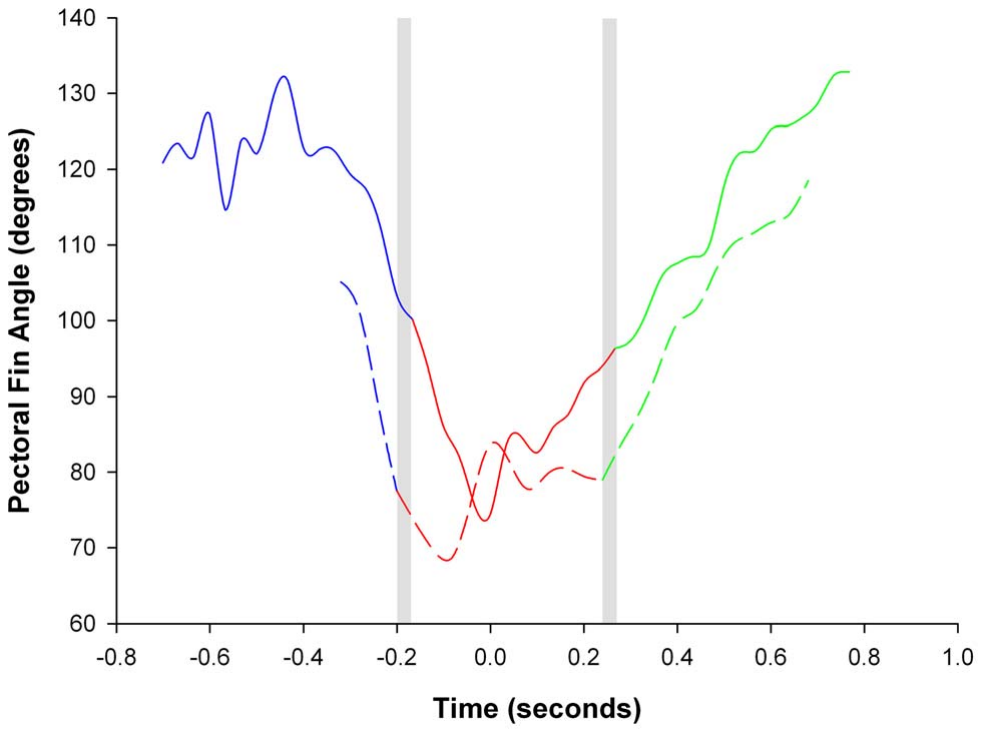
The adduction and abduction angles of a thresher shark's pectoral fins during overhead tail-slaps. Angles for transverse plane events (n = 2) were measured for preparation (blue); strike (red); and wind-down recovery (green) phases, and are aligned from the point at which the tips of the tail reached their maximum height (x/y intercept  = *0*).

Preparation phases were characterised by thresher sharks lunging at the bait ball in the horizontal plane (mean 10.36±4.80° SD). Lunging never resulted in prey capture but was usually followed by a tail-slap. As a thresher shark accelerated into a lunge, there was little vertical movement of the tracked anatomical parts (Figure 8-A), and the pectoral fins remained obtusely orientated (120°–129°) to each other (Figure 9). The mean (± SE) duration of the preparation phases was 1.04±0.17 seconds (95% CI: 0.71–1.37 seconds). The duration of a preparation phase and size of shark were not correlated (f_1,14_  = 3.13, p = 0.099). Although lunge speeds could not be measured, there was no qualitative difference between the preparation phases of the analysed overhead tail-slap events.

Strikes were always preceded by lunges and often resulted in prey capture. The mean (± SE) duration of the strike phase was 0.39±0.01 seconds (95% CI: 0.36–0.41 seconds). The duration of a strike phase and size of shark were not correlated (f_1,14_  = 0.07, p = 0.795).

To initiate a strike, a thresher shark first lowered its snout and flexed its body dorso-ventrally, causing the caudal-peduncle and tail to dip and tension (Figures 4, 5, 8-A). The shark then adducted its pectoral fins to an acute orientation (74° in relation to each other) (Figures 5, 9). The manoeuvre changed its pitch to a mean (± SD) camber of −32.49°±9.26° promoting its posterior region to lift rapidly, and stall its lunge approach. After stalling its approach an abrupt lateral rotation of the pectoral fins prevented the shark's posterior region from continuing to precipitate forward. The caudal peduncle then flexed ventro-dorsally causing the tip of the tail to accelerate vertically in a trebuchet catapult motion and be slung overhead (Figures 4, 5). The trajectory of the tip of the tail reached its apex above the dorsal fin. It then travelled the rest of the length of the shark's body to its terminal point above the tip the snout, which was slightly raised by the mechanics of the behaviour (Figure 8-A).

**Figure 10:**
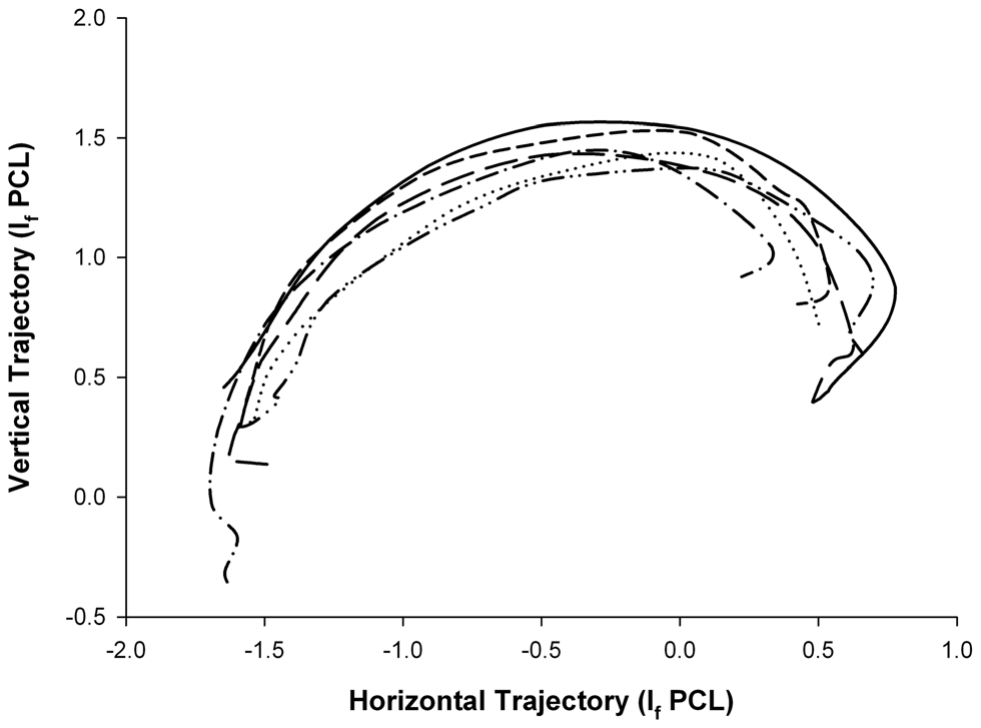
Comparative analysis of the trajectory arc that formed the path of a thresher shark's overhead tail-slap as observed from six hunting events recorded in the sagittal plane. The relative distances (cm) the tip of the tail was from the posterior base of the pectoral fin (x/y intercept  = *0*) were plotted at intercepts that were standardised for precaudal length. The motion of the trajectory is left to right.

A two-way analysis of variance found no significant differences between the shapes of the trajectory that formed the path of a thresher shark's tail-slap among the six events that were recorded in the sagittal plane (f_5,40_  = 1.04, p = 0.409). Figure ten shows the extent to which the trajectory paths were similar when standardised for precaudal length (Figure 10).

During a strike, the mean (± SE) speed with which the tip of the tail travelled over a thresher shark's body was 14.03±1.01 ms^−1^ (95% CI: 12.05–16.01 ms^−1^) for all sharks combined (n = 16). Tail-slap speeds were biphasic and would accelerate to their maxima, and then decelerate until the tip of the tail reached its terminal point above the snout (Figure 8-B). The time it took for the tip of a thresher shark's tail to reach its maximum speed was not different from the time it took to reach its maximum height (t_5_ = 0.66, p = 0.537). A tail-slap's maximum speed was therefore achieved at the apex of the arc that formed the path of its trajectory (Figure 8-C).

**Figure 11:**
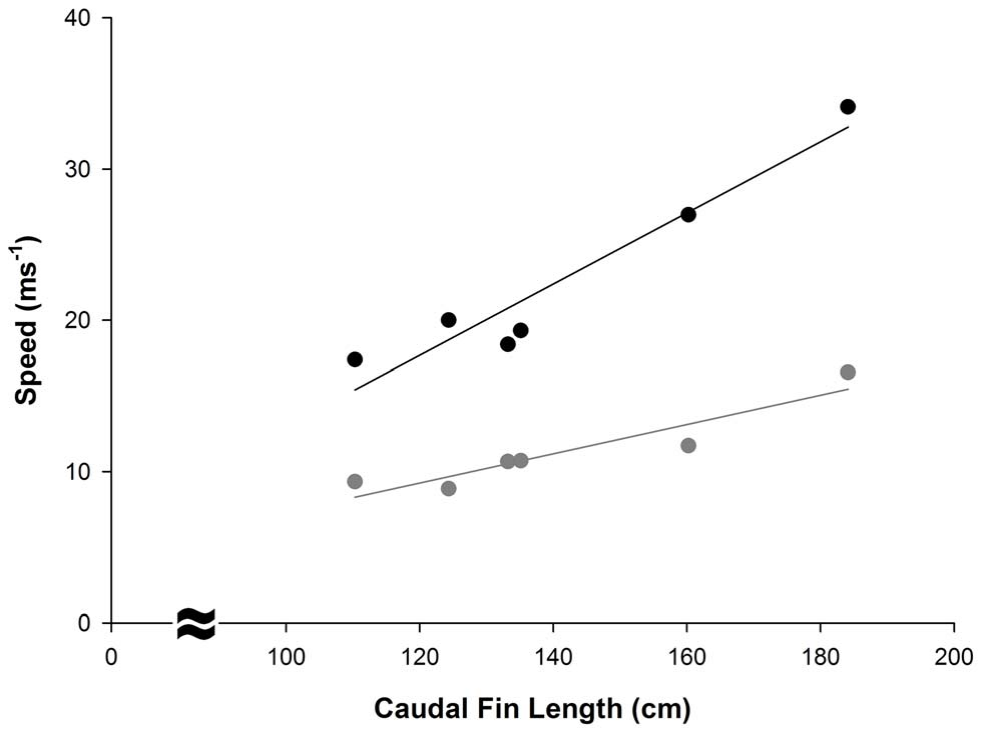
Relationship between the mean (grey) and maximum (black) tail-slap speeds and the length of a thresher shark's tail. Speeds are expressed in meters per second (ms^−1^), and are regressed against the caudal fin (CDM) lengths of individual sharks that were observed in the sagittal plane (n = 6).

During three of the successful events, bubbles were observed to form where the tip of the tail reached its maximum speed and height (Figure 4, Movie S1). The fastest tail-slap, which had a mean speed of 21.82 ms^−1^, resulted in prey capture and the formation of a plume of bubbles. The slowest tail-slap, which had a mean speed of 8.86 ms^−1^, did not result in prey capture or the formation of bubbles. The mean speed with which a thresher shark slapped its tail was directly related to its size and the length of its tail (PCL: f_1,4_  = 17.28, p = 0.001; CDM: f_1,4_  = 26.94, p = 0.007) (Figure 11). Since frame-by-frame analysis of video sequences could only be achieved for recordings of the overhead tail-slaps that were observed in the sagittal plane, maximum tail-slap speeds could not be calculated for all sharks. When considering only the six sagittal plane events, a significant relationship was found between the maximum speed with which a thresher shark slapped its tail, shark size, and tail length (PCL: f_1,4_  = 52.18, p = 0.002; CDM: f_1,4_  = 46.6, p = 0.002) (Figure 11). However, a tail-slap's mean rotational speed was not related to a thresher shark's size or the length of its tail (mean PCL: f_1,4_  = 0.00, p = 0.979; mean CDM: f_1,4_  = 0.1, p = 0.767; max PCL: f_1,4_  = 0.37, p = 0.577; max CDM: f_1,4_  = 0.33, p = 0.593).

Wind-down recovery phases, during which the snout, caudal peduncle and tail were returned to their original condition, always began when the tip of the tail reached the terminal point of its trajectory above the snout (Figures 4, 5). During a wind-down recovery phase, the snout and caudal-peduncle were returned to a horizontal position, and the pectoral fins were abducted to their original orientation of 120°–129° relative to the underside of a shark's body (Figures 4, 5, 8-A). The caudal fin was then returned to a vertical position in the sagittal plane, where it began to oscillate laterally for locomotive purposes. The mean (± SE) duration of the wind-down recovery phase was 0.51±0.05 seconds (95% CI: 0.41–0.61 seconds). The duration of a wind-down recovery phase and size of shark were not correlated (f_1,14_  = 0.52, p = 0.483).

**Figure 12:**
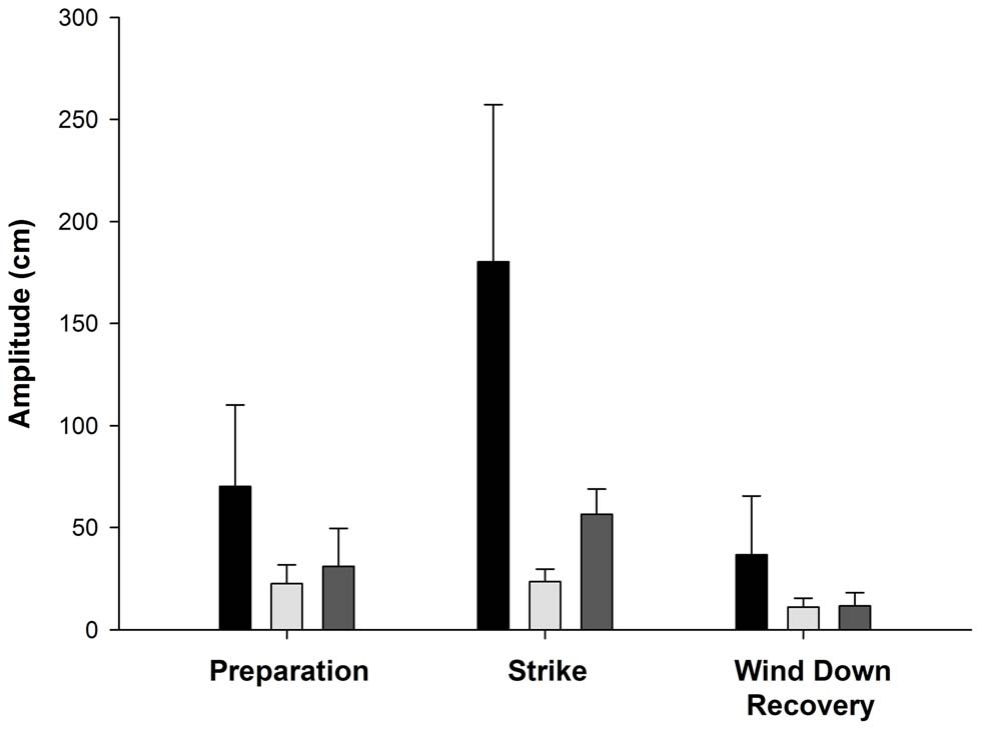
Amplitudes of a thresher shark's caudal fin (black), caudal peduncle (light grey) and snout (dark grey) movements during preparation, strike and wind-down recovery phases.

The relative amplitudes of the tracked anatomical parts varied among preparation, strike and wind-down recovery phases (anatomical part: f_2,45_  = 28.89, p<0.001; phase: f_2,45_  = 20.99, p<0.001; part*phase interaction: f_4,45_  = 8.01, p<0.001) for all events (Figure 12). Figure 12 shows how distinct the amplitudes of the tracked anatomical parts were for each of the phases.

### Sideways Tail-Slaps

Although SCUBA divers observed sideways tail-slaps *in situ* on several occasions during field observations, they were only recorded on videotape three times (Movie S4). Recorded sideways tail-slaps either occurred at a distance from the camera, or were partially obscured by the sardines, and were therefore only used for qualitative descriptions of the behaviour.

Sideways tail-slaps were always preceded by a successful overhead tail-slap and took place during an ongoing prey item collection phase of a thresher shark's predation attempt. However, not all overhead tail-slaps were followed by sideways tail-slaps. Preparation, strike and wind-down recovery phases could not be delineated from the video records, even though they were observed *in situ.* The mean (± SE) duration for sideways tail-slaps was 5.23±1.45 seconds (95% CI: 2.39–8.07 seconds). The longest recorded sideways tail-slap took place over 7.90 seconds and the shortest lasted 2.93 seconds.

**Figure 13:**
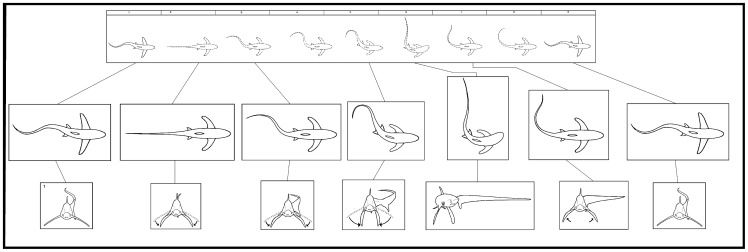
Behaviour diagram of a thresher shark's sideways tail-slap, with preparation (1, 2), strike (3–6) and recovery (7–9) phases, as observed in the sagittal plane. A motion animation (top) represents 4.86 s^−1^ of an event which was recorded by handheld underwater video camera on 14 June, 2010. Center inserts profile the key characteristics of the behaviour, while inserts shown in the transvers plane (bottom), were interpreted from *in situ* observations.

During sideways tail-slaps, there was little vertical movement of the snout, caudal peduncle or tail. First a thresher shark positioned itself alongside the schooling prey fish. Then the pectoral fins were adducted and a strike was initiated by flexing the trunk and caudal peduncle laterally. The tail was then whipped laterally to one side of a thresher shark's body. The trajectory of the tip of the tail followed a horizontal path, which terminated in line with the first dorsal fin. The snout, caudal peduncle and tail were then returned to their original condition (Figure 13).

## Discussion

While it has long been suspected that thresher sharks hunt with their tails, little was previously known about the behaviour in the wild. This study represents the first attempt to quantify the kinematic patterns associated with alopiid predatory behaviours in their natural environment, and implies that adaptive foraging techniques play an important role in the hunting strategies of large marine predators.

### Recorded Events

Thresher sharks are physiologically adapted for thermo-tolerance and demonstrate distinct crepuscular vertical migrations [30] by spending their days well below the thermocline (200–700 m), and their nights in surface waters (0–200 m) [31]. Patterns of vertical movements in sharks can reflect foraging, thermoregulation, predator avoidance, energetics and reproduction behaviours [32], [33]. Since thresher sharks are nocturnally active and feed primarily on small fish and cephalopods, their vertical movements at night are presumed to be related to their hunting strategy [27], [28]. Yet in contrast to what was known previously, observations of pelagic thresher sharks at Pescador Island occurred as they hunted during daylight hours.

Anecdotal evidence suggests that pelagic thresher sharks circumnavigate the surface waters of the Philippines at all times of the day. The sharks regularly visit cleaning stations by day [28], and they are taken as by-catch by fishermen targeting sardines in the morning after sunrise. It is possible that pelagic thresher sharks pursue sardines opportunistically, by day and night, which may make them vulnerable to fisheries.

During overhead tail-slaps, preparations were significantly longer in duration than strike and wind-down recovery phases. The lunge acceleration, which characterises a preparation phase, may position a thresher shark within reach of the sardines it is preying upon to facilitate stunning them [3]. Lunges may also generate enough forward momentum to aid in the lift of a thresher shark's posterior region, by providing a windup speed for a pectoral adduction manoeuvre to counter. Since significantly more time is needed to windup the tail for a strike than its release and wind-down recovery, preparations are longer in duration than the other primary phases.

After the wind-down recovery phase, almost all of the observed thresher sharks turned 180°, presumably to search for and collect dead and/or stunned sardines. One third of the overhead tail-slaps resulted in prey items being collected, and when successful, a thresher shark consumed more than one sardine. Carnivorous oceanic sharks generally pursue one prey item at a time [7], [12], [34], [35]. Prey capture is however, infrequent compared to the time spent searching, and energy expenditure can be high since many prey items are elusive [11], [34]–[36]. It has been suggested that large marine predators are particularly inefficient chase feeders when they prey upon small schooling fishes [37]. To overcome the confusing defense mechanism of fish schooling, predators have had to adopt different hunting strategies [38], [39].

While the manoeuverability of small schooling fish can make individuals elusive to predators chasing them individually [3], they become vulnerable in numbers to tail-slaps adapted to hunt them, and the shockwaves associated with the behaviour [2], [3], [40]. Being able to stun more than one prey item at a time is efficient since it enables a thresher shark to increase the likelihood of it gaining a substantial cost/benefit reward even though its predation attempts are only successful a third of the time.

Bubbles were observed to form at the apex of an overhead tail-slap's trajectory during most of the observed successful thresher shark hunting events (Figure 4, Movie S1). During a tail-slap, rapid changes of pressure in the water may occur locally, due to the acceleration of flow around the leading edge of the caudal fin [41]. At sufficient velocity the pressure around the leading edge may be so high that the corresponding turbulent pressure may drop below the saturated vapour pressure [42], [43], causing dissolved gas to diffuse out of the water column into small bubbles that entrain and grow in size [39], [44]–[46].

### Tail-Slap Kinematics

While the sample size of the hunting events that met the selection standards for analysis was too small to quantify stereotypy, tail-slaps were remarkably invariable among trials for different sharks. To launch a strike, a thresher shark first adducted its pectoral fins, a manoeuvre that changed its pitch promoting its posterior region to lift rapidly (Movie S2). A shark's attack camber then peaked between −30° and −45°, well above the known stalling threshold that occurs in a hydrofoil suggesting that overhead tail-slaps occurred at least in part, under stalling conditions [3], [47]. Because the bait ball at Pescador Island does not move much in the water column, it may be preferable for a thresher shark to attack it under stalling conditions, rather than risk causing the sardines to disperse from the school in a flight response by continuing to lunge at them.

Immediately after adducting its pectoral fins, a thresher shark rotated them laterally to counter the momentum of its posterior region from precipitating forward. Wilga and Lauder (2000, 2001) described the vertical movements of leopard (*Triakis semifasiata*) and bamboo (*Chiloscyllium plagiosum*) sharks, being initiated by acute angular adjustments of their aplesodic pectoral fins, which generate negative and positive lift [48], [49]. In contrast, thresher sharks possess plesodic pectoral fins that are straight, broad-tipped and long, with radials extending far into their fin webs [7]. In addition to being specialised for fast cruising [50], a thresher shark's pectoral fins may be ideally adapted to control its pitch and rotational momentum during a tail-slap.

The analyses of the video records showed that the dorsal margin of a thresher shark's caudal fin, which is flexible and narrows to the tip [7], [8], [17], [28], contributed most to the angular excursion of a tail-slap. When investigating the hunting strategies of the common thresher shark *Alopias vulpinus*, Aalbers et al. (2010) reported a dominant “tail-feeding strategy” that thresher sharks initiated with a “forward undulation of the anterior body, which resulted in a posterior-travelling sinusoidal wave that consequently advanced along the body towards the uppermost tip of the caudal fin”. In pelagic thresher sharks the movement driving a tail-slap did not appear to be a rapidly propagated wave of action potentials that advanced along a shark's body, but rather the result of a rapid and powerful ventro-dorsal peduncular movement that drove the tail from its base in a trebuchet catapult motion.

Although a tail-slap's rotational speed was invariable among different sharks, the mean and maximum speeds of a tail-slap were directly related to a thresher shark's size. In physics, the ‘kinetic link’ principle implies that during a catapult launch motion, energy and angular momentum are transferred from one body segment to another, in a sequential manner, all the way to the distal segment [51], [52]. If two distal segments of different lengths are rotated at the same angular speed, the longer one will travel faster. The muscular and vertebral segments of a thresher shark's body that are sequentially involved in the tail-slapping process increase in size and length throughout a shark's ontogeny [53]–[55]. Larger sharks with longer tails are likely to require greater strength to execute a tail-slap than smaller conspecifics because they have more mass to accelerate. When considering the dorsal caudal fin margin as the distal segment involved in the tail-slapping process, having more strength to drive a larger mass at the same rotational speed advantages bigger sharks because their longer tails will strike at prey faster.

### Sideways Tail-Slaps

From their investigations of common thresher sharks Aalbers et al. (2010) described a second predominant strike behaviour during which a shark “positioned itself in close proximity and parallel to the prey item before initiating a lateral strike of the dorsal lobe”. Yet at Pescador Island, pelagic thresher sharks' sideways tail-slaps were rare, only occurring after successful overhead tail-slaps, when sardines had already been stunned (Movie S4).

During sideways tail-slaps, thresher sharks were observed to target single prey fish, which swam erratically and were not elusive, presumably because they had been previously stunned and/or injured. It is possible that sideways tail-slaps are a specialist technique to debilitate maimed prey further for consumption.

There were occasions during *in situ* observations when two or more thresher sharks sideways tail-slapped different areas of the bait ball at approximately the same time suggesting social behaviour. From surface observations of white sharks (*Carcharodon carcharias*), Klimley et al. (1996) described tail slapping as an agonistic threat signal between two conspecifics competing for the same food resource. A shark was said to only be able to feed on a dead seal floating in the vicinity “if the vigor and frequency of its tail-slap were greater than its opponent” [22]. Other studies have shown that large marine predators will hunt socially, using their tails to control the shape and density of bait balls [1]–[3], [26], and it has been speculated that thresher sharks use the technique to corral schooling prey [7], [13]. Since field reports of two or more thresher sharks deploying sideways tail-slaps concurrently at Pescador Island were anecdotal and unverified, proposals that the behaviour was an agonistic signal among conspecifics over resource competition, or that they hunted cooperatively were treated with caution.

### Conclusions

The evidence is now clear; thresher sharks really do hunt with their tails. Tail-slaps comprise four distinct phases that sequentially function to windup, strike, and recover the tail, and if successful, collect stunned prey items. Tail-slapping is an efficient strategy for hunting schooling prey since thresher sharks are able to consume more than one prey item at a time. Larger thresher sharks tail-slap faster than smaller ones because their tails are longer. A thresher shark's pectoral and caudal fins appear to have evolved, at least in part, to deploy tail-slaps. Analyses of the peduncular, radialis and axial musculature, which are likely to be recruited for the execution of a tail-slap, as well as the changes in kinetic energy of the fins and body relative to a thresher shark's centre of mass, would help to elucidate the motor patterns driving the kinematics of this unusual hunting behaviour in future studies.

## Supporting Information

Movie S1 **Overhead tail-slap with preparation, strike, wind down recovery and prey item collection phases.** Recorded in the sagittal plane on 24 August 2010, cavitation bubbles can be seen rising from the apex of the arc forming the trajectory of a pelagic thresher shark's strike. This event resulted in the shark successfully debilitating and consuming three sardines. Video material provided by Jan Acosta (© 2010).
(M4V)

Movie S2 **Overhead tail-slap with preparation, strike, wind down recovery and prey item collection phases.** Recorded in the transverse plane on 9 September 2010, this successful event resulted in a pelagic thresher shark debilitating and consuming two sardines.
(M4V)

Movie S3 **Overhead tail-slap with preparation, strike, wind down recovery and prey item collection phases.** Recorded in the sagittal plane on 24 May 2010, this event did not result in prey capture and was classified as unsuccessful.
(M4V)

Movie S4 **Sideways tail-slap.** Recorded in the sagittal plane on 14 June 2010, this event followed a previously successful overhead tail-slap. Sideways tail-slaps appeared slow and lazy compared to overhead tail-slaps.
(M4V)
